# Sexual harassment against female nurses: a systematic review

**DOI:** 10.1186/s12912-020-00450-w

**Published:** 2020-06-29

**Authors:** Woldegebriel Gebregziabher Kahsay, Reza Negarandeh, Nahid Dehghan Nayeri, Merzieh Hasanpour

**Affiliations:** 1 Department of Community Health and Geriatric Nursing, School of Nursing and Midwifery, International Campus, Tehran University of Medical Sciences (IC-TUMS), Tehran, Iran; 2 Department of Midwifery, College of Medicine and Health Sciences, Adigrat University, Adigrat, Tigray Ethiopia; 3 Nursing and Midwifery Care Research Center, School of Nursing and Midwifery, Tehran University of Medical Sciences, Nosrat St., Tohid Sq., Tehran, Postal code: 1419733171 Iran; 4 Department of Critical Care Nursing and Nursing Management, School of Nursing and Midwifery, Tehran University of Medical Sciences, Tehran, Iran; 5 Department of Pediatrics Nursing and Neonatal Intensive Care, School of Nursing and Midwifery, Tehran University of Medical Sciences, Tehran, Iran

**Keywords:** Female nurses, Health consequences, Perpetrators, Prevalence, Sexual harassment

## Abstract

**Background:**

Sexual harassment is complex and has occupational hazards in nursing. Nurses experienced it than other employees. Female nurses are with the highest rate in the profession. Our aim was to determine the prevalence of sexual harassment against female nurses, the types, perpetrators, and health consequences of the harassment.

**Method:**

We undertook a systematic review to synthesize quantitative research studies found in Pubmed, Scopus, ProQuest, Web of Science and Google Scholar databases. The studies included were observational, on sexual harassment against female nurse, full text, and published in peer-reviewed English journals up to August 2018. Two independent reviewers searched the articles and extracted data from the articles. The quality of the articles was evaluated using the Modified Newcastle Ottawa Scale for Cross-Sectional Studies Quality Assessment Tool. A descriptive analysis was done to determine the rate of items from the percentages or proportions of the studies.

**Result:**

The prevalence of sexual harassment against female nurses was 43.15%. It ranged 10 to 87.30%. The 35% of the female nurses were verbally, 32.6% non-verbally, 31% physically and 40.8% were being harassed psychologically. The 46.59% of them were harassed by patients, 41.10% by physicians, 27.74% by patients’ family, 20% by nurses and 17.8% were by other coworker perpetrators. The 44.6% of them were developed mental problems, 30.19% physical health problems, 61.26% emotional, 51.79% had psychological disturbance and 16.02% with social health problems.

**Conclusion:**

The prevalence of sexual harassment against female nurses is high. Female nurses are being sexually harassed by patients, patient families, physicians, nurses, and other coworkers. The harassment is affecting mental, physical, emotional, social and psychological health of female nurses. It is recommended policymakers to develop guidelines on work ethics, legality and counseling programs. Nursing associations to initiate development of workplace safety policy. A safe and secure working environment is needed in the nursing practice and nursing curriculum in prevention strategy. Research is needed on factors associated with sexual harassment. Since only female nurses were the participants, it could not be representative of all nurses. There was no fund of this review.

## Background

Sexual harassment and violence against nurses is complex and also became occupational hazards in the nursing profession. This happened to the opposite of the professional mission to care who appears to be at the highest risk of workplace violence [1]. Nurses are exposed to experience the offensive behaviors at work than other employees [2]. Since the job brings the nurses physically and emotionally close to patients and other staff members, they are with the highest rate of sexual harassment in the profession [3]. One in forth nurse worldwide reported exposure to sexual harassment [2].

Even though upwards of 90% of nurses are female, nurses still experience sexual harassment from their co-workers and colleagues [4]. In addition to this, the other sources of sexual harassment are patients, patients’ families, and visitors who account for some harassment [1, 5]. A report showed that female nurses were more sexually harassed by patients than male nurses, 73% for female nurses and 46% for male nurses respectively [6]. The prevalence of sexual harassment by patients was also high, with 60% of female nurses worldwide reporting the incident [2, 7].

Research on sexual harassment in the workplace is in its infancy, but according to the European Union, 40–50% of women experienced sexual harassment or unwanted sexual behavior in their workplace [8]. The report on the global supply chain showed that 85% of female employees were concerned with sexual harassment [9]. In a field dominated by women, nurses are particularly susceptible to sexual harassment in the workplace [10]. In a study, 91% of nurses reported experiencing at least one type of sexual harassment, 30% experienced more than three and about 5% reported on five or more types of sexual harassment [11]. However the female nurses’ aspect is little known.

Sexual harassment is an unwelcome and offensive conduct of a sexual nature that may make workers feel humiliated, intimidated or uncomfortable [9]. It is unwelcome sexual advances, requests for sexual favors, and other verbal or physical conduct of a sexual nature that is directed toward a person in the workplace [12].

Sexual harassment has also taken many forms [9]. It may include unwelcome verbal, visual, nonverbal, or physical conduct that is of a sexual nature or based on someone’s sex [13]. The Physical form of harassment is unwelcome touching, fondling, hugging or kissing. Verbal form of harassment includes sexually suggestive, offensive, comments or jokes; inappropriate invitations to go out on dates; intrusive, offensive questions about private life; intrusive, offensive comments about a woman’s physical appearance. Non-verbal forms is inappropriate, intimidating, staring or leering; receiving or being showed offensive, sexually explicit pictures, photos or gifts; indecent exposure; being made to watch or look at pornographic material against one’s wishes. The last is the use of technological cyber harassment faced by receiving unwanted, offensive, sexually explicit emails or SMS messages; inappropriate, offensive advances on an internet website or in an internet chat room [9].

In the sexual harassment, a perpetrator is a harasser who may be a woman or a man [14]. Therefore, the perpetrator of sexual harassment in this study would be any of the male or female gender around the female nurses’ working area. According to the World Health Organization (WHO), the perpetrators of harassment and violence may be persons in positions of authority who are respected and trusted such as physicians [10]. Data also showed that sexual harassment is a demonstration of personal power over others [15]. A qualitative study showed that physicians were at the top of hierarchy as perpetrators and the nurses at middle level of hierarchy [3]. In addition to this, the prevalence of sexual harassment committed on nurses was 82% by physicians, 20% by coworkers and 7% by immediate supervisors were accounted for most incidents [5]. In another online survey, 5% out of 749 female nurses had experienced sexual harassment by another staff member including physicians in past 3 years of the study [16].

Sexual harassment can affect individuals in a number of ways, including their mental and physical health, finances, and opportunities to advance in their careers [13]; victims of sexual harassment can suffer significant psychological effects, including anxiety, depression, headaches, sleep disorders, weight loss or gain, nausea, lowered self-esteem and sexual dysfunction [17]. Any of those would be considered as the health consequences of sexual harassment in this review.

Though increasingly sexual harassment and violence are considered as important occupational safety and health issues, it is largely invisible and unreported [9]. This is especially true considering that many nurses fail to report incidents of harassment, no matter who was at fault. Many nurses have developed a thick skin, and are used to the “sexy nurse” stereotypes that doctors, patients and other nurses may impose on them. The reasons for non-reporting sexual harassment are complex and multifaceted but typically include fear of retribution or ridicule, and a lack of confidence in investigators, police and on other health workers [10]. In addition to this, many hospitals overlook harassment done by their most accomplished physicians, even reported [4, 5]. The sexual harassment by co-workers as well as patients were also an issue that has received considerably less attention than physical and nonphysical violence [2].

There is an abundance of research papers and qualitative reviews on sexual harassment against nurses in general, to our knowledge there are no quantitative reviews specifically on female nurses. Most studies and reviews were focused on non-nurse women [18]. Studies among nurses were with a mix of male and female nurse participants and student nurses [2]. It is of great importance to examine the prevalence, types of sexual harassment, perpetrators, and health consequences of sexual harassment to female nurses. On the part of the victim, it may help in understanding sexual harassment more clearly and inform policymakers, get priority attention and for its protective measures. Female nurses are a graduated nurse assigned to provide care for healthy or ill clients. Prevalence is the percentage of female nurses that faced sexual harassment.

### Objectives

This systematic review was to determine the prevalence of sexual harassment against the female nurses, types of sexual harassment, perpetrators, and its health consequences on female nurses working in hospitals reviewing observational studies.

#### Research questions

What is the prevalence of sexual harassment against female nurses, what are the types of sexual harassment, who are the perpetrators, what is the health consequence of sexual harassment on female nurses from observational studies?

## Method

### Review protocol

The Preferred Reporting Items for Systematic Reviews and Meta-Analysis 2009 (PRISMA 2009) guideline was followed to report in this systematic review [19]. Electronic searches were completed on 30 August, 2018 of the complete databases Scopus (from 2004), Google Scholar (from Nov. 2004), Pubmed (from June 1997), Web of Science (from 1997) and ProQuest (from1972). This extended time and suitable database use were to get adequate information.

All studies globally conducted on sexual harassment against female nurses were searched and used in the analysis. The systematic search was from suitable databases to identify potentially eligible articles for the analysis. To screen eligible articles, inclusion criteria were set. A retrieve of statistics from the articles was done and descriptive analyses were conducted. Analyses began by computing the weighted mean to pool the percentage or proportions of female nurses’ exposure to sexual harassment with respect to prevalence, types of sexual harassment, perpetrators and health consequences of sexual harassment on female nurses from the articles.

#### Eligibility criteria

We searched for studies on sexual harassment. The eligible articles included in the review study were: Observational studies on female nurse participants, full text, published in peer-reviewed English journals, concerning prevalence of sexual harassment, and on female nurses who were graduated and working in any health facility to provide care for well or ill clients. But qualitative studies, reviews, and abstracts were excluded. Since the outcome is sexual harassment against female nurses, studies among male gender or any mix of male and female nurses as participants were excluded. Student nurses and other women in non-nursing employment were also excluded.

#### Information sources

The comprehensive literature search was focused on sexual harassment against female nurses. Studies were identified by searching out the following electronic databases: PubMed, Scopus, ProQuest, and Web of Science and Google Scholar. All searches were limited to the English language in scholarly journals and full text articles.

#### Search strategy

The Electronic searches were done to get potentially eligible studies. The following key terms were used in each database. From the advanced Google Scholar search was “sexual harassment OR sexual OR violence OR sexual OR assault “female nurses” limited to words occur anywhere in the article. The advanced search on Pubmed was “((((([sexual) AND [violence]) OR [harassment]) OR [assault])” limited to full free articles and studies on females. The search on ProQuest was “ti (sexual harassment) OR ti (sexual violence), ti (sexual assault) AND ti (female nurses)”. On Scopus was “sexual AND harassment AND violence OR assault AND female AND nurse”. On Web of Science “TI= (sexual AND harassment)” OR “TI=(sexual AND violence)” OR “TI=(sexual AND assault)” AND “TI=(female AND nurses)” and combination of terms was used.

#### Study selection

**Fig. 1:**
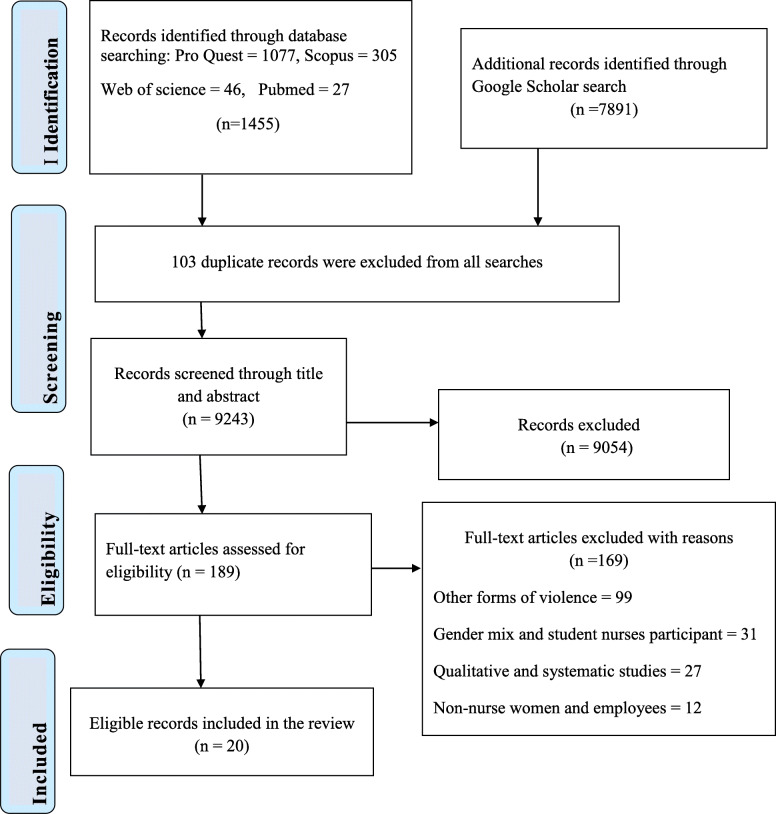
PRISMA flowchart search results on sexual harassment against female nurses. Adapted from Liberati et al., 2009 [19]

The search identified a total of 9346 records. During the initial screening, 103 duplicate records were removed. Out of the 9243 records, 9054 records was excluded based on the title and the abstract. Afterwards, full-text articles (*n* = 189) were independently reviewed if they met the inclusion criteria for this systematic review by two reviewers. Out of the 189 articles, excluded articles were: 99 articles on other forms of violence, 27 articles qualitative and review in design, 31 were with gender mix and student nurse in participants, 12 dealt on other health care providers and other women employee participants. Finally, a total of 20 articles was found eligible for this systematic review. The PRISMA flow chart was used in the selection as shown in Fig. 1 [19]. Discrepancies in article selection of the two reviewers were resolved by discussion. For issues with disagreement, resolved by a third reviewer.

#### Data collection process and data items

Before the synthesis of the primary studies, the documents were read thoroughly to gain an initial sense of the data by two authors. The two authors independently identified items with their percentages and recorded in a tabulated data sheet. The data sheet includes authors’ name and year of publication, study country, study design, the sample size of female nurses, items for type sexual harassment with their forms, perpetrator list with its percentage and items of health consequences of sexual harassment with their forms were identified. The first and second authors independently examined the included studies and the first author extracted the relevant data, which was cross-checked by the second author. For issues with disagreement, resolved by a third reviewer.

#### Risk of bias in each study

The two reviewers independently reviewed each article of its quality. The quality of the articles was evaluated using the Modified Newcastle Ottawa Scale for Cross-sectional Studies Quality Assessment Tool before the analysis. The tool has ordinal scoring for the following components: the representativeness of the samples, sample size, non-response rate, and use of a validated tool, comparability, outcome and statistical test. Each component is rated as 9–10 points of very good study, 7–8 points for good studies, 5–6 points for satisfactory studies and 0 to 4 points for unsatisfactory studies in quality [20]. These articles, which scored satisfactory or more (≥ 5 scores) in quality were included in this systematic review. The two reviewers scored a rating for each article. A third reviewer was involved solving the disagreement between the two reviewers in the rating of study quality. A final agreed-upon rating was assigned to each study (Table 1).

**Table 1:** Prevalence, types and forms of sexual harassment against female nurses

Authors and Year	Country	Sample size	Prevalence	Types and forms of sexual harassment	Quality Score (0–9)
Othman et al. 2018, [21]	Kenya	120	27.50%	Sexual harassment	**5**
Fatema 2017, [22]	Bangladesh	133	71.00%	Sexual harassment	5
Hussein et al.2015, [23]	Egypt	110	87.30%	Verbal forms: Verbal comments to 27.57%,, Talking sexual words to 3.03%, Saying sexual jokes to 3.3%, Sending telephone to 12.43% Non-verbal: Whistling to 11.2%, sex look to 38.17%, Waving to 5.13%, Forced identification to 15.17%, Stalking to 2.7%, Touching nurses’ body to 4.23%, Removal of clothes to 2.70%	7
Ali et al.2015, [24]	Egypt	430	70.20%	Staring in suggestive manner at 70.90% of female nurses, Talking by sexual words to 58.60%, Comments or jokes to 57.30%, Unnecessary touches to 49.30%, Making an intercourse offer to 30.5%,Threatened sex to 9.6%, Rape to 1.30%	7
Mushtaq et al. 2015, [25]	Pakistan	200	71.66%	Sexual harassment	6
Khan et al.2015, [26]	Pakistan	150	80%	Sexual harassment	5
Park et al. 2015, [27]	South Korea.	970	19.70%	Sexual harassment	6
Subedi et al.2013, [28]	Nepal	134	40.30%	**Verbal:** Vulgar words heard by 22.39%, Vulgar jokes heard by 35.82%, **Visual**: People staring at 14.93% nurses’ body People showing naked pictures to 2.24% **Contact**: Trying to touch 4.48% of nurses Embracing without permission to 18% Blackmail for sex to 2.24%, Threatening for sex to 2.24% Facing rape like situation to 2.24%	5
Suhaila & Rampal 2012, [29]	Malaysia	455	51.20%	Verbal sexual harassment to 46.60%, Visual harassment to 24.80% Psychological harassment to 20.90%, Physical harassment to 20.70% Non –verbal harassment to16.70%	6
Shiao et al. 2010, [30]	Taiwan	842	28.10%	Sexual harassment, Physical Harassment,Verbal Harassment Assault 3.5%	7
Hibino et al. 2009, [31]	Japan	464	56%	Sexual jokes to 64.30% of the nurses, Physical contact to 59.70%, Gazing with sexual interest to 36.70%,Request for dating to 27.20%, Request to touch patients body to 14.80%, Hugging to14%, Stalking to 9.80%, Rape to 0.90%	7
Celik & Celik 2007, [32]	Turkey	622	37.10%	**Sexual Harassment** Uninvited sexual jokes, stories, questions, or words to 11.62% Allusive sexual behaviors with the eye, hand, or face to 6.98% Unwillingly asked out to 6.1%, Unwanted mail or telephone calls to 5.14%, Touched on the body to 4.22%, Perpetrators shown their body sexually to 3.98%, Any attempt to assault to 0.18%	7
Gunnarsdottir et al. 2006, [33]	Iceland	600	10%	Sexual harassment to 10%	8
Hibino et al. 2006, [34]	Japan	464	55.80%	**Verbal**: Making sexual jokes and remarks to 12.28%, Asking about private matters to 5.39%, Asking for dating in 3.23%, Making threats to 2.16% **Non-verbal**: Gazing at nurses to 4.74% Stalking or visiting the nurse at her home to 1.08% **Contact**: Touching the nurse’s body to as hugging to 10.78%, Trying to touch nurses’ body in 10.78%, Requesting the nurse to touch the patient’s body, Trying to bring the nurse to the patient’s bed, Attempting to sexually assault the nurse to 0.43%	8
Kisa et al. 2002, [35]	Turkey	215	73%	Suggestive looks to 20%, Sexual teasing and jokes to 19%, Suggestive physical gestures to 18%, Pressure for dates to 14%, Unwanted letters, telephone calls to 10%, Exposure of parts of the, body in a sexually suggestive way to 11%, Brushing, touching or grabbing to 19.53%	5
Matsuoka et al.2001, [36]	Japan	243	49.4%	Touching to 5.35%, gossip to 3.3%, brought up the topic of sexual relations to7.82% Asking sexual relations to 5.35%, touch body to 5.35, tried to touch nurses body in 24.28%, sexual jokes or words to 17.28% comment on body to 13.58%, gazing in an unpleasant manner to 6.17%, Send nude photo to 2.47%, send letter/phone call containing sexual issue to 3.29%	6
Shaikh 2000, [37]	Pakistan	89	43.67%	Verbal sexual harassment to 21.10% Physical sexual harassment to 16.90%	5
Kisa & Dziegielewski 1996, [38]	Turkey	229	75%	Sexual teasing and jokes to 72%, Suggestive physical gestures to 65%, Pressure for dates to 53%, Unwanted letters, telephone calls to36%, Exposure of parts of the body in a sexually suggestive way to 40%, Brushing, touching or grabbing and grossly inappropriate touching to 26.64%	7
Dan et al.1995, [39]	United States	52	80%	**Sexual harassment** Suggestive stories or offensive jokes to 51.9%, Unwelcome seductive behavior to 46.1%, Unwanted sexual attention to 41.4% Deliberately touched and made uncomfortably to 41.67% Unwanted discussion of personal or sexual matters to 35.87%	5
Libbus & Bowman 1994, [40]	United States	78	71.80%	Sexual jokes to 13.46%, Sexual remarks to 19.23%, Touch (brushing, patting, hugging) to 14.74 19%	5

**Note:** The study designs were descriptive cross sectional in all articles

#### Synthesis of results

A descriptive analysis was done to get weighted mean of the percentages or proportions of the prevalence, perpetrators, each type of sexual harassment and health consequence items. Prevalence rates for studies were calculated as weighted means. The prevalence rate per study was multiplied by the corresponding sample size and divided by the total sample size of all studies. The results were summarized using descriptive statistics for this systematic review.

Types of sexual harassment and the consequences of sexual harassment were grouped into categories. The percentages and proportions of the items were pooled.

## Results

### Study characteristics

The studies included in this systematic review were 20 [21–40]. All of them were conducted on sexual harassment against female nurses in descriptive cross-sectional designs. The countries for the studies conducted in were: Four studies (20%) in Pakistan, two (10%) in Turkey, three (15%) in Japan, two (10%) in the United States, two (10%) in Egypt. And these seven countries contributed one study (5%) each: Malaysia, Nepal, Taiwan, Iceland, Kenya, Bangladesh, and South Korea. The total participants were 6600 (Table 1). All the studies reported the prevalence of sexual harassment and the types of sexual harassment or their forms. Fifteen (75%) studies reported at least one or more perpetrators. For the consequences of sexual harassment, fifteen (75%) studies reported one or more symptoms or health consequences of sexual harassment to female nurses (Table 2).

**Table 2:** Perpetrators and health consequences of sexual harassment on female nurses

Author/Year	Perpetrators	Consequences of sexual harassment
Othman et al., 2018 [21]	–	Headache on 57.5%, fatigue on 56.67%, difficult sleep on 54.17%, Nightmare on 48.33%, and loss of appetite on 35% of nurses Stomach pain, weight gain, weight loss, disturbances of the menstrual cycle, muscular spasm or convulsions, and gastric ulcer disease or hypertensive.
Fatema, 2017 [22]	–	Feeling of sadness on 54%, loss of self-confidence on 25%, crying for no reason on 35% and Social isolation on 22%. Uncontrolled ferociousness on 19%, trouble in emotional relationships on 27%, and bitterness on 48%.
Hussein et al., 2015 [23]	43.30% patient /family 30% follow nurse 26.70% doctors	Anxiety (Mean 41.27 ± 6.12) z = 3.85, *p* = 0.000 Depression (Mean 33.40 ± 4.44) z = 2.10, *p* = 0.036
Ali et al., 2015 [24]	42.70% patients 61.90% patients’ family 12.90% doctors 45.40% staff	Felt anger on 37.10% Felt shame on 30.40% Psychological in general on 94.7% Disappointment on 76.50% Depression on 67.90%, and fear on 35.80%
Mushtaq et al. 2015	–	Depression, anxiety, stress
Khan et al.,2015 [25]	55.3% patients/visitors 25.3% physicians 4.7% colleague nurses 14.7% administration	Psychological in general on 50.7% Physical health in general on 8%
Park et al., 2015 [27]	55.5% patients 15.2% patient family 34.6% physicians 2.6% colleague nurses 1.6% nurse managers	–
Subedi et al., 2013 [28]	18.52% patients 25.93% patient relatives 37.03% physician 11.11% administrative staff	–
Suhaila & Rampal, 2012 [29]	Patients Patients’ relatives Colleagues Medical Officers	Psychological effects in general on74.70%. Fear on 80.30%, depression on 26.6%, loss of appetite on 8.60%, nausea on 7.70%, and fatigue on 1.30%.
Shiao et al., 2010 [30]	Psychiatry patients	Night shift had negative effects on the score of general health (Coef-6, SE = 2.7 p = 0.03) Working in a psychiatric hospital was positively associated with scores in mental health (Coef 2.7,SE = 0.6 *p* < 0.0001) Working in psychiatry hospital was vitality, was negatively associated with these scores (Coef 1.7,SE = 0.7 *p* < 0.0110)
Hibino et al., 2009 [31, 34]	Patients	–
Celik & Celik 2007 [32]	43.30% patients 34.20% attendants 77.10% physicians 51.10% nurses 29.4% other personnel	Disturbed mental health on 44.6% Physical problem in general on 24.20% Sleeping difficulty on 24.20%, headache on 40.30%, stomach ache on 17.30%, negative social and family relations on 36.80%, disturbed family life on 27.30%, being tired on 14.30%, fear on 23.4%, helplessness on 17.30%, depression on 10.80%, belittlement or humiliation on 10.8%.
Gunnarsdottir et al., 2006 [33]	–	Psychological wellbeing affected
Hibino et al., 2006 [31, 34]	94% male patients	–
Kisa et al., 2002 [35]	39% patients 17% relatives of patient 41% physicians 4% other hospital staffs	Emotional reactions -Anger on 42.9%, fear on 11.4%, helpless on 8.2%, depression on 6.9%, feelings of humiliation on 10.5%, guilt/self-blame on 6.2%. Physical symptoms: Headaches on 37.7%, Dizziness on 2%, gastritis on 12.9%, nausea and/or vomiting on 2.4%, exhaustion on 12.9%, menstrual disturbances on 2%, inability to sleep on 20.2% and sleep more on 3.2%.
Matsuoka et al., 2001 [36]	–	Mental problem on 41%.
Shaikh, 2000 [37]	2.80% male patients 11.27% male attendants 26.90% male physicians	–
Kisa & Dziegielewski,1996	34% patients 14% relatives of patients 44% physicians 9% other perpetrators	Emotional effects -Anger on 44%, feelings of humiliation on 14%, fear on 12%, guilt on 9%, and depression on 5% and helplessness on 5%. physical symptoms: Headaches on 38%, inability to sleep on 20%, feelings of exhaustion on 15%,gastritis on 14%, nausea and/or vomiting on 4%, tendency to sleep more than usual on 1%, dizziness on 1%, and menstrual disturbances on 1%.
Dan et al.,1995 [38]	75% patients 73% visitors 88.5% physicians 83% coworkers	Emotional condition on 47.4%. Physical condition on 11.1%.
Libbus & Bowman,1994 [40]	53.5% patients 5.4% patients’ family 25% physicians 12.5% non-nurse staff 3.6% nurses	Emotional responses on 70.5% (Anger on 23.6%), embarrassment on 19.6%, disgust on 19.6%, nervousness on 18.20%.

#### Prevalence of sexual harassment

The prevalence of sexual harassment against female nurses ranged from 10% [33] to 87.30% [23]. The pooled prevalence was 43.15%. The types of sexual harassment were verbal, non-verbal, psychological and physical in a sexual nature.

The 35% of the female nurses (ranged 21.1 to 46.6%) faced with verbal sexual harassment [23, 29, 30, 37]. Among the forms of verbal sexual harassment, 42.33% of participants (ranged 3.03 to 58.60%) heard bad words with sexual matters [23, 24, 28], and 25.45% (ranged 3.3 to 72%) heard bad jokes on sexual matters [23, 28, 31, 32, 34–36, 38–40], and 37.8% of participants (ranged 13.58 to 57.3%) faced with sexual comments or remarks [23, 24, 36, 40]. Again, in the verbal form of harassment, 21.33% of participants (ranged 3.23 to 53%) were being asked for prospective partner relationship [31, 35, 38]. The 8.45% of female nurses were being asked their private matters ranged 3.39 to 35.87 [34]. The 5.9% of the participants (ranged 5.35 to 6.11%) were being asked for sexual relation unwillingly [32, 36]. About 7.82% were invited a topic on sexual relations for discussion [36]. In addition to this, 10.34% of participants received unwanted mail/blackmail or telephone calls for sexual relation the rate ranged from 5.14 to 36% [23, 28, 32, 35, 36, 38].

One-third (32.6%) of the female nurses was harassed in non-verbal type of visual sexual harassment [29]. In the forms of visual sexual harassment, 18.06% of female nurses (ranged 4.74 to 36.7%) were harassed in a sexual suggestive look [31, 34–36]. About 38.17% were faced in an unwanted sexual attention [23] and 57.67% of participants (ranged 14.95 to 70.9%) were in the form of staring at nurses’ body [24, 28]. The 19.89% (ranged 2.7 to 65%) were harassed in facial expression forms [23, 32, 35, 38]. The perpetrators removed their clothes and showed their body’s sexuality or naked picture 9.8%, it ranged 2.24 to 40% [23, 28, 32, 35, 36, 38].

In respect of the physical sexual harassment type, 31% of participants (ranged 11.64 to 59.7%) were harassed physically [29–31, 37]. The 13.68% of participants (4.48 to 24.28%) tried to be touched their body by perpetrators [28, 34, 36], and 11.04% of nurses’ body unnecessarily and without their permission were touch (ranged 4.22 to 41.67%) [23, 24, 28, 31, 32, 34, 35, 38–40]. The 8.23% of participants requested to touch patient’s body [31]. To 0.65% of the participants, patients tried to bring the nurses to their bed [34]. The 1.24% of the participants (ranged 0.9 to 2.24%) were raped [24, 28, 31] and the 1.71% were faced sexual assault, the rate ranged 0.16 to 3.56% [30, 32, 34].

The 40.8% of the female nurses were faced with psychological type of sexual harassment in one study [29]. About 5.28% were threatened for sex, ranged 2.16 to 9.6% [24, 28, 34]. The 30.55% were pressured for an intercourse [24]. About 41.4% got unwanted sexual attention and 46.1% were exposed to unwanted seductive behavior [39]. About 3.3% were exposed to gossip [36], and the 7.72% in stalking form of harassment, the rate ranged 1.08 to 9.8% [23, 31, 34], 11.2% were exposed to whistling behavior [23], and 15.17% were for forced identification [23] (Table 1).

#### Perpetrators of the sexual harassment against the female nurses

The perpetrators of sexual harassment against female nurses were patients, patients’ families or visitors, physicians, nurses, and other coworkers. The 46.59% of the participants (ranged 2.8 to 94%) were harassed by patients in 12 studies [23, 24, 26–28, 32, 34, 35, 37–41]. About 27.74% of participants harassed (ranged 5.4 to 73%) by patients’ family in 9 studies [24, 27, 28, 32, 35, 37–41]. About 41.12% harassed by physician perpetrators, the rate ranged from 12.9 to 88.5% in 11 studies [23, 24, 26–28, 32, 35, 37–40]. The 20% were harassed by nurses, the rate ranged 2.6 to 83% in five studies [23, 26, 27, 32, 40]. About 17.8% of participants were by other coworkers and staff and ranged from 1.6 to 45.40% in eight studies [24, 26–28, 32, 35, 38].

#### Health consequences of sexual harassment on female nurses

The health consequences of sexual harassment identified with this review were mental, psychological, emotional, physical and social health consequences. The 42.8% of female nurses developed mental health problem [32]. Anxiety was one of the mental problems with a mean score of 41.27 points [23]. The 16.76% of the victims (4.02 to 47.67%) had depression [24, 29, 32, 35, 38] with the mean score level of 33.4 in its severity [23, 25].

Nearly one-third (30.19%) of the female nurses had physical health problem due to sexual harassment, the rated ranged 8 to 37.14% [26, 32, 39]. Regarding symptoms of the physical consequences, 27.8% had a headache, the rate ranged from 14.95 to 57.5% [21, 32, 35, 38], 10.2% felt exhaustion, the rate ranged from 0.66 to 56.67% [21, 29, 32, 35, 38] and the 1.56% had dizziness ranged between 0.87 and 2.33% [35, 38].

The gastrointestinal tract related consequences were 10.79% of nurses lost their appetite, the rate ranged between 4.4 and 35% [21, 29]. About 13.33% had increased their appetite [21]. Nearly 4% female nurses had nausea or vomiting, the rate ranged between 2.79 and 4% [29, 35, 38]. The 13.51% diseased with gastritis, the rate ranged between 12.9 and 14% [35, 38], and 11.59% of participants had stomach ache, the rate ranged between 6.43 and 38.34% [21, 32]. The 14.17% of nurses had weight gain and on the other hand 29.6% had weight loss [21].

Moreover, 48.33% had nightmares [21], about 17.79% had sleep difficulty, the rate ranged between 9 and 54.17% of female nurses [21, 32, 35, 38]. The 7.22% of participants (ranged 0.87 to 20%) had slept long [21, 35, 38]. The 8.51% of nurses had menstrual disturbances, the rate ranged between 2.33 and 27.5% [21, 35, 38]. Nearly 16% felt muscular pain or convulsed as physical health consequences [21].

More than half (61.26%) of the female nurses were emotionally disturbed by sexual harassment, the rate ranged from 47.4% [39] to 70.5% [40] in two studies. In the emotional consequences, 29.51% became anger and nervous, the rate ranged 16.67 to 50.22% [24, 35, 38, 40]. About 21.56% felt fear and ferociousness, the rate ranged 8.68 to 41.1% in the seven studies [22, 24, 29, 32, 35, 38, 40]. The 16.84% of female nurses (ranged 53.72 to 54%) were disappointed or felt sad [22, 24]. The 16.36% of the participants cried without reasons (35%) ranged 10.6 to 35% [22, 24] and 48% of the nurses felt sense of bitterness [22]. About 20.28% of participants (ranged 14.10 to 21.40%) felt shame and embarrassment feelings [24, 40]. Nearly 9% of the participants (ranged 4.02 to 15.72%) had feelings of belittlement and humiliation [32, 35, 38].

More than half (51.79%) of nurses (ranged 38.24 to 66.53%) were psychologically disturbed as in three studies [24, 26, 29]. In the psychological suffer, 9.91% felt guilt or self-blame, the rate ranged from 8.84% [35] to 10.92% [38]. The 19.6% had disgust [40], 25% lost their self-confidence [22] and the 7.41% felt helplessness, the rate ranged 6.11% [38] to 11.63% [35].

There were also social health consequences to the female nurses due to the sexual harassment. The 16.02% of nurses had social disturbance, the rate ranged 13.67 to 27% [22, 32]. Again, 17.33% of the nurses had family life disturbance ranged 10.13 to 51% [22, 32], and social isolation in 22% of participants [22] (Table 2).

## Discussion

The result of this systematic yield 43.15% of female nurses sexually harassed and it ranged from 10 to 83.5% in prevalence. This high prevalence is similar with previous studies resulted in 16 to 76% of nurses sexually harassed [42], and 53.7% of female nurses that perceived being harassed [43], 60% of nurses harassed [44], 63.6% of nurses in another study [45], and 66% of nurses and nurses students face the harassment [46]. However, it is lower than the 91% of nurses and nurse students sexual harassed in medical centers [11]. This difference could be due to the mix in male and female nurse participants who had different roles of nurses and nurse students.

The study also indicated that female nurses were faced with multiple types harassments related to their sexual nature verbal, non-verbal, physical and psychological types of sexual harassment in their workplace. This result agrees with the types of sexual harassment experienced in health care workers [47].

The verbal type of sexual harassment happed to female nurses in different forms. Many of the female nurses heard bad words of sexual matters, bad jokes related sexual issues and the perpetrators were forwarding comments in a sexually manner. This shows similarity with other studies among nurses, nurse students and female graduates out nursing that faced verbal forms of sexual harassment [5, 11, 45, 48, 49]. The comment form of verbal harassment was lower in magnitude than the comments against nurses and student nurses [11]. This difference could be due to differences in study participants. In addition to this, the female nurses were harassed verbally as in unwanted mail/blackmail or telephone calls for sexual relations, asked their private matters, asked for a sexual relationship unwillingly, initiated for unnecessary sexual relations and were being asked for prospective partner relationship. There is similarity in these forms and magnitude with those female graduates got sexual messages posted on notice board, other got text messages for relation, asked to do something sexual in exchange for favors [5, 49]. But the magnitude is lower than the study among nurses and student nurses that faced romantic relation [11]. This difference could be due to difference in participants of being male and female nurses and nurse students.

The study also indicated that non-verbal types of sexual harassment experienced in a significant number of female nurses. In a visual form, the female nurses had faced a suggestive sexual look or gazing at sexual interest and had been forced to see body sexuality or a naked picture of perpetrators. These forms are in keeping with nurses and nurse students faced leering or ogling and as in perpetrators showed or left sexual pictures to female graduate students [46, 49, 50]. This is significantly high harassment which could interfere with the nurses’ day to day duties.

According to this review, a number of female nurses were also physically harassed. The physical type of the sexual harassments were in different forms. The female nurses’ body was tried to be touched by perpetrators and the female nurses’ body unnecessary and without their permission was also touched. It is consistent with other studies results as unnecessary and unwanted touches to nurses and other female graduates, intimated touching of nurses’ body, unnecessary touching, patting, or pinching of body parts of nursing students, and female graduates and nurses forced to kiss someone or to do something sexual other than kissing [11, 46, 49, 50]. In addition to the touch type of harassment, female nurses were raped, forced attempt on intercourse, faced sexual assault, requested to touch the patient’s body and patient perpetrators tried to bring female nurses to perpetrator’s bed. It is in keeping with the rape to employees, forced attempt on intercourse against nurses, and student nurses faced a proposition to the intimate relationship [11, 44, 51].

In this systematic review, we found that female nurses were affected by the psychological type of sexual harassment in their workplace. One form of the psychological sexual harassment was a sexual threat to the female nurses. The result agrees with the nurses pressured for sexual cooperation [5]. To provide compassionate nursing care, female nurses should get the right to humanistic, peaceful and care related relationship with people stay in hospitals and other medical centers.

This review identified a number of different perpetrators in the sexual harassment against the female nurses. The first most and highest in rank of harassment against the female nurses was from clients; the 46.59% of female nurses were harassed by patients. Its rank and its range for the rate is consistent with other similar study results by patient harassers to 72.8% of nurses [45], 62.9% nurses [48], to other result of 58% nurses [44], and to 18–38% nurses and nurses students harassed by patients [11, 46]. The second most source of harassment was by physicians. The 41.12% of female nurses were harassed by physicians. The rank and its rate agree with the 10–30% of nurses and nurse students harassed [11], and 57.9% of nurses in other study [45].

About 27.74% of participants were harassed by patients’ family. This is different result compared with the 3% nurses harassed by visitors [46]. The difference could be due to the participant difference gender of participants and the study design. The 20% of female nurses were harassed by nurses. This result is consistent in rank and the rate of the range with the 15–22% nurses and nurse students [11] and 13% of nurses harassed by nurse [44]. Most of the harassers are stayed in the female nurses’ working place health facilities. Therefore, there should be system design in hospitals and other medical centers to bide the harassers in creating safe working environment for the female nurses.

The result of this review showed that sexual harassment against the female nurses resulted in mental, physical, emotional, psychological and social health consequences. The consequences generally agree with workplace aggression consequences such as fear, anxiety, disappointment, and being helplessness on nurses and nurse students [52]. Again, shows similarity with the study reported the consequences of sexual harassment among nurses of the mental and physical health consequences in their workplace [48].

Mental health problem was one of the consequences of sexual harassment resulted in the female nurses. It is keeping with the workplace violence consequence of nurses [51]. The review identified the forms mental health consequences as anxiety, depression and stress. This finding show an agreement with other studies reported anxiety, depression and stress after sexual harassment to nurses and nurse student and female graduate students [5, 27, 49, 52].

The review indicated that sexual harassment against the female nurses led them to have the physical health consequences. The consequence manifested in the form of headache, exhaustion and for gastrointestinal disturbances gastritis, nausea or vomiting, weight gain or weight loss, neuromuscular problems such as muscle pain or convulsion and dizziness. These multiple manifestations get similarity with other study among nurses and nurses students [5, 18, 44, 46, 50], In addition to these manifestations, the female nurses had other physical manifestation sleeping difficulty, had inability to sleep, and others abnormally slept long. This is in keeping with nurses, nurse students and female graduates students sleep disturbances [43, 49, 50]. However, headache is higher in magnitude compared to other study result [46]. This difference could be the participants’ gender mix and its study design in that study.

In this review study nearly half of the female nurses emotionally disturbed due to the sexual harassment against them. This result agrees with the health consequence happened to health care workers [47]. It also indicated that forms of emotional disturbances felt by female nurses were becoming anger and nervousness, fear or become ferocious, had feeling of disappointment or sadness, shamefulness or embarrassment and feeling of humiliation and belittlement. It shows agreement with the study on nurses, nurse students and health worker harassment health consequences [18, 44, 46–48, 50, 52, 53]. But the magnitude of feeling of shamefulness, embarrassment, humiliation and belittlement were low compared to study results among nurses and nurses students working in medical centers [11]. The difference could be the gender mix and mix in roles of participants in that study.

The systematic review indicated that a significant number of female nurses was psychologically affected in their health due to the sexual harassment. They lost their confidence, become helpless, disgusted, had suffered from self-blame. It shows agreement with a study on behavioral consequences of sexual harassment on nurses, nurses and nurse students and other employees [11, 18, 46, 47, 52]. These all consequences happening to the female nurses are unexpected and unaccepted additional burden of the professionals which demand counseling service to the victims.

## Limitations

This literature review provides an overview of knowledge on sexual harassment against female nurses. However, this review covers the articles only published in English, the reviewed articles were cross-sectional design. Most of the rates were calculated from a few study results and participants were only female who may not be representative of the nurse population.

## Conclusion

According to this review, the prevalence of sexual harassment against female nurses is high and persisting in magnitude in the nursing profession. The types of sexual harassment include physical, verbal, non-verbal and psychological with their different forms in a sexual nature. First ranked perpetrators are the service demanding clients. The second next perpetrators are physicians that were assigned to improve the quality of health. Next third perpetrators are patients’ family. The fourth ranked perpetrators of sexual harassment are nurses. The rest were other coworkers. Female nurses are being affected mentally, physically, and emotionally, socially and their psychology due to the sexual harassment.

This is the time policymakers to develop guidelines on work ethics, legal accountability, team work and counseling programs to manage and reduce the consequences of sexual harassment among being affected female nurse. The nursing associations are recommended initiating female nurses’ workplace safety policies and strategies in hospitals to minimize this tradition in the profession. Health managers are recommended to create a safe and secure working environment for female nurses which contributes in improving the quality nursing care. Female nurses to create unity which able them to identify, prevent, minimize the occurrence of harassment and manage each consequence at spot in their working hospitals. Nursing curriculums to include sexual harassment prevention strategies and improve life skills of female nurses in tackling sexual harassments. Researchers to find out technology for information, communication and reporting systems of sexual harassment. It is also recommended investigating the factors associated with sexual harassment against the female nurse and use predictive research designs.

## Data Availability

The datasets used during the current study are available from the corresponding author on request.

## Abbreviations

NICU: Neonatal Intensive Care Unit
PRISMA: Preferred Reporting Items for Systematic Reviews and Meta-Analysis
WHO: World Health Organization
