# Correction to: Sexual harassment against female nurses: a systematic review

**DOI:** 10.1186/s12912-020-00456-4

**Published:** 2020-07-13

**Authors:** Woldegebriel Gebregziabher Kahsay, Reza Negarandeh, Nahid Dehghan Nayeri, Marzieh Hasanpour

**Affiliations:** 1 Department of Community Health and Geriatric Nursing, School of Nursing and Midwifery, International Campus, Tehran University of Medical Sciences (IC-TUMS), Tehran, Iran; 2 Department of Midwifery, College of Medicine and Health Sciences, Adigrat University, Adigrat, Tigray Ethiopia; 3 Nursing and Midwifery Care Research Center, School of Nursing and Midwifery, Tehran University of Medical Sciences, Nosrat St., Tohid Sq, Tehran, 1419733171 Iran; 4 Department of Critical Care Nursing and Nursing Management, School of Nursing and Midwifery, Tehran University of Medical Sciences, Tehran, Iran; 5 Department of Pediatrics Nursing and Neonatal Intensive Care, School of Nursing and Midwifery, Tehran University of Medical Sciences, Tehran, Iran

**Correction to: BMC Nurs 19, 58 (2020)**

**https://doi.org/10.1186/s12912-020-00450-w**

Following publication of the original article [1], the authors identified an error in the author name of Marzieh Hasanpour.

The incorrect author name is: Merzieh Hasanpour

The correct author name is: Marzieh Hasanpour

The author group has been updated above and the original article has been updated.
